# Supplementary figures and images for: Basis profile curve identification to understand electrical stimulation effects in human brain networks
Source: PLoS Comput Biol. 2021 Sep 2;17(9):e1008710. doi: 10.1371/journal.pcbi.1008710 (PMC8412306; doi:10.1371/journal.pcbi.1008710)

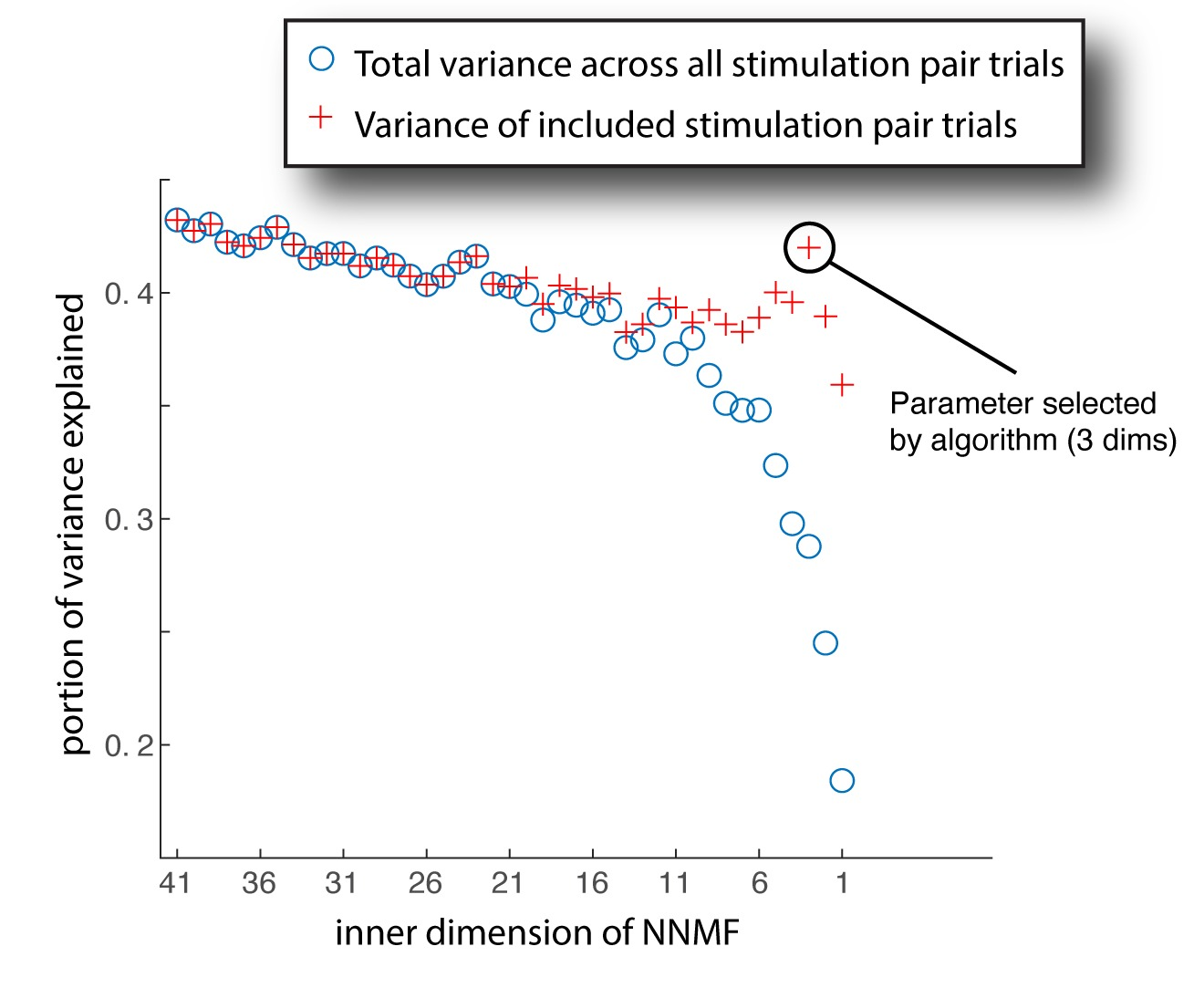

Supplement: S1 Fig — We can quantify the total variance explained by the fitting of resulting BPCs, Bq(t), across all included stimulation-pair trials: 〈Vk⊤Vk−εk⊤εk〉k∈n∈q. When we normalize this by the total variance, 〈Vk⊤Vk〉k∈alln, we have the portion of total variance explained (blue circles). However, the goal of the decomposition is to identify motifs, explained by BPCs, from the full set of stimulations. For example, if one were to perform stimulations at a site that has no effect on the measurement site, it should not undermine our confidence in the decomposition. Therefore, a more appropriate normalization is to instead divide the explained variance by the variance of the trials included in the clustering, 〈Vk⊤Vk〉k∈n∈q. For our example case, this immediately validates the dimensionality Q = 3, selected by the algorithm (Fig 3). (TIF) [file pcbi.1008710.s001.tif]

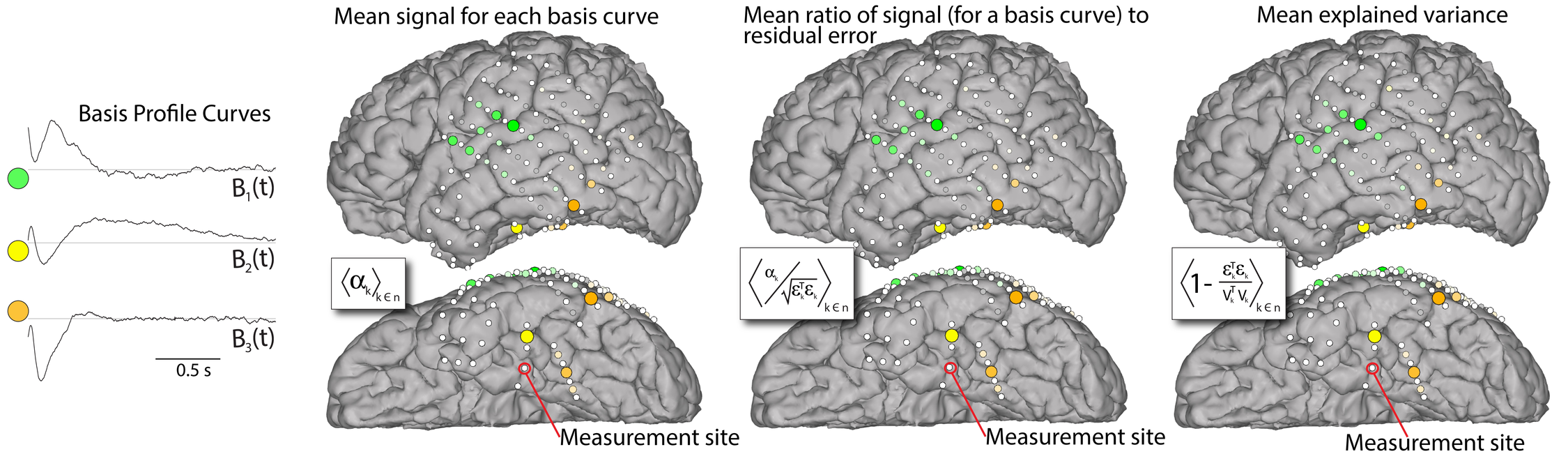

Supplement: S2 Fig — As in Fig 4, but using alternate scoring metrics for each stimulation-pair subgroup, shown in inset rectangles overlying each cortical rendering. (TIF) [file pcbi.1008710.s002.tif]

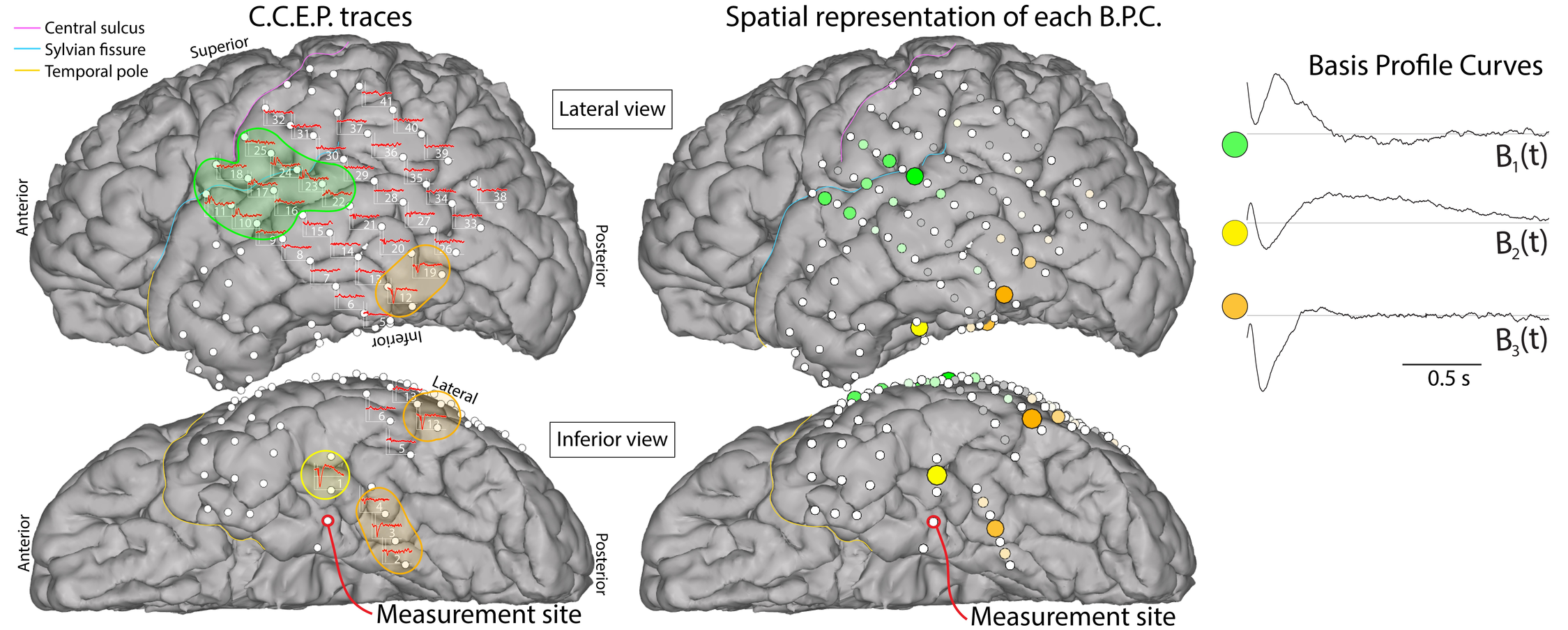

Supplement: S3 Fig — (TIF) [file pcbi.1008710.s003.tif]

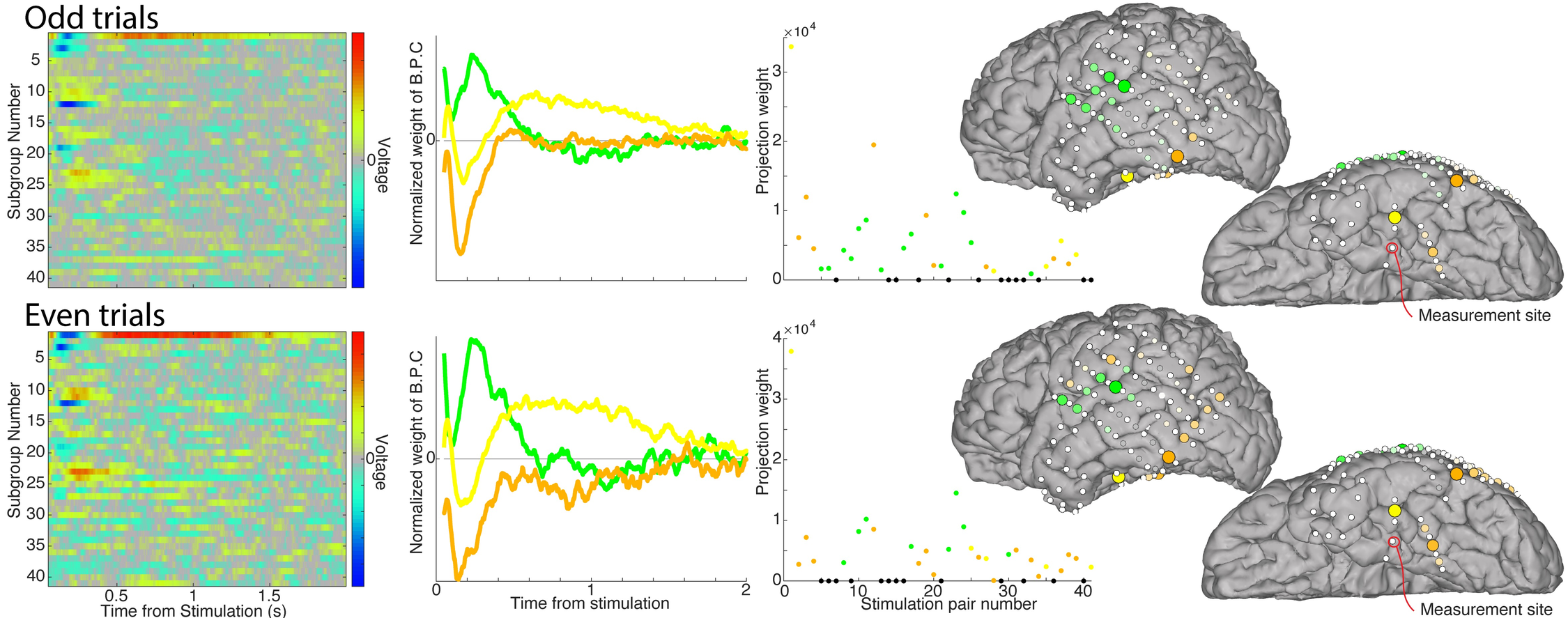

Supplement: S4 Fig — On the top row, BPCs are identified from only odd trials. On the bottom row, BPCs are identified from only even trials. After the split, each stimulation-pair subgroup is represented by 4–5 individual stimulation trials. Despite this small amount of individual stimulation trials for each stimulation-pair subgroup, the process is remarkably stable. For each row, the panels from left-to-right are matrices of the CCEPs, extracted BPC shapes, projection weights (group-averaged signal-to-noise ratio), and projection weights plotted on the brain surface. (TIF) [file pcbi.1008710.s004.tif]

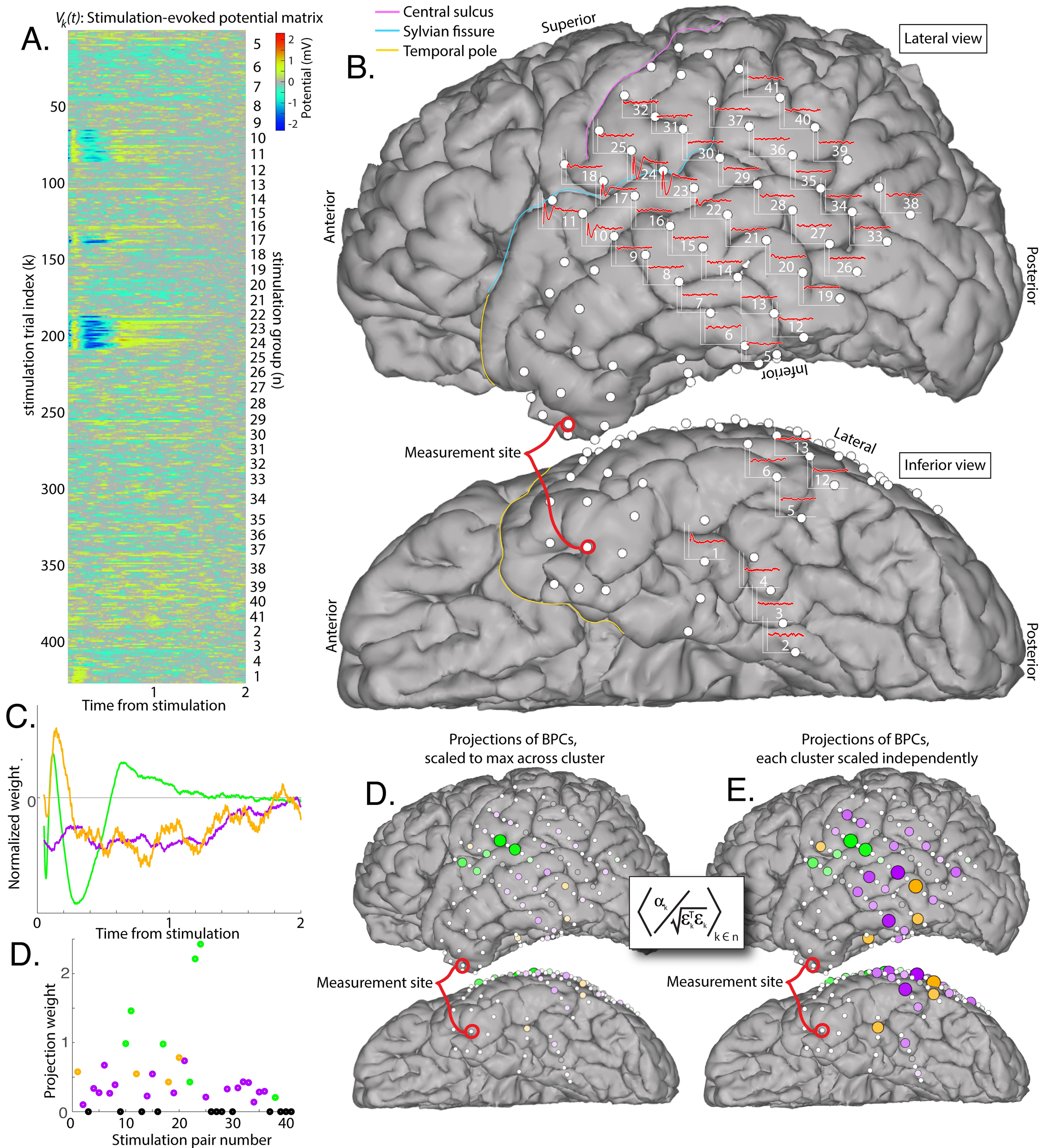

Supplement: S5 Fig — Stimulation responses from a site in the temporal pole are shown. A: Responses from each stimulation pulse are aligned into a matrix Vk(t). B: Averaged responses Gn(t) are shown at the site of each stimulation pair that produced them. C: BPCs produced by the algorithm. D: Weights (group-averaged signal-to-noise ratio) associated with each BPC (color-coded), and non-included sites (gray). E: Spatial representation of BPCs, color-coded, with diameter and color intensity indicating magnitude. All values are scaled to the global maximum across all BPCs. F: As in (E), but with each BPC distribution individually scaled to its own maximum. Note the similarity in the spatial distribution of the stimulation-site cluster labeled in green to Fig 3), but the completely different shape of the BPC (likely reflecting the different laminar architecture of the two recipient measurement sites). (TIF) [file pcbi.1008710.s005.tif]
