# Supplementary material for: Basis profile curve identification to understand electrical stimulation effects in human brain networks
Source: PLoS Comput Biol. 2021 Sep 2;17(9):e1008710. doi: 10.1371/journal.pcbi.1008710 (PMC8412306; doi:10.1371/journal.pcbi.1008710)
Supplement: Attachment — Submitted filename: kjm_bpcmethod_reply_to_reviewers_R1.pdf [file pcbi.1008710.s006.pdf]

Mayo Clinic

Department of Neurosurgery
200 First Street SW
Rochester, Minnesota 55905

June 24, 2021

Dear Editors of *PLOS Computational Biology*,

This is a reply to reviewers for the manuscript "**Basis profile curve identification to understand electricalstimulation effects in human brain networks**" (PCOMPBIOL-D-21-00124), by Miller, Müller, and Hermes.

We are very grateful for the thoughtful comments and suggestions from each of the reviewers. We feel that they have greatly improved the manuscript. All comments have been directly addressed, and the manuscript has been modified in response to each one. There were many modifications in the revision, but all were to specifically improve the manuscript in response to reviewer concerns.

We have made an unsolicited change to the manuscript and the framework it describes, replacing the global cost-function in the NNMF dimensionality reduction (sum of off diagonal elements), Greek letter zeta, with a maximum pair-wise cost function (maximum of off diagonal elements). This has proven in our subsequent work to be a more stable, reliable, quantity.

In this reply to reviewers document, gray text indicates commentary from the reviewer, **black** text indicates author response, and blue text indicates quotation from the manuscript. When required, LaTex format has been retained in responses.

Sincerely,

Kai J. Miller, Mayo Neurosurgery, on behalf of Klaus-Robert Müller, Dora Hermes, and myself

miller.kai@mayo.edu; Cellular phone (USA): +1 206 355 0244

Reviewer #1:

1. The principal question I have is related to how this technique performs relative to straightforward existing methods, such as comparing evoked responses.
   a. Do the BCP derived curves shown in Fig 3 and then again in Fig 4 look like ERPs from the different stimulation sites?
   Yes, it is correct that the BPCs shown in Figures 3 and 4 look like the recorded cortico-cortical evoked potentials (CCEPs) from the different stimulation sites. We have added a new supplemental figure that shows these side-by-side for simple comparison. This is referenced in the text of the results: "The technique produces an intuitive representation of the responses measured at a single site in the convergent paradigm (Supplemental figure 3)."

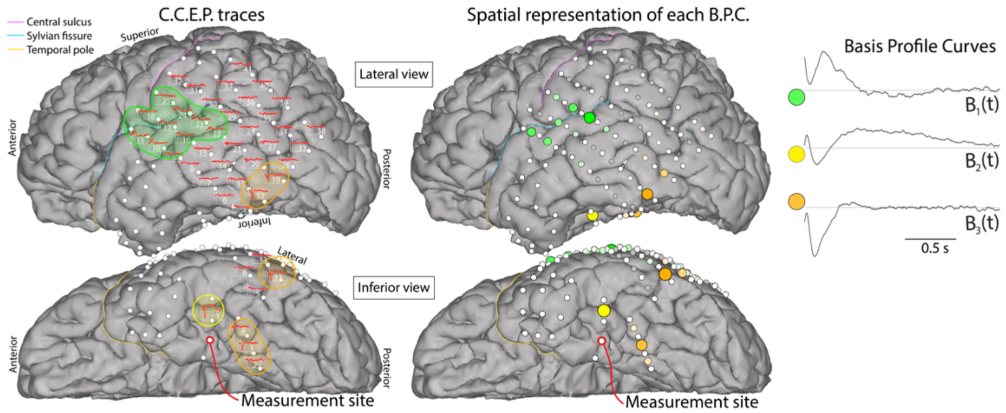

Supplemental Fig 3. Side-by-side illustration of CCEP shapes (from Figure 2E) and BPC projection weights (from Figure 4B&C), to illustrate the correspondence.

   b. Could the same discernments in connectivity motifs arise from existing methods, such as examining N1 and N2 components or measuring correlations in the ERPs for example?

   We find that N1 and N2 components are not universal, as we note in the introduction, which we have further clarified in a now-modified sentence: "Notably, we have found that the N1/N2 response shape is not a universal phenomenon: when observed, this shape is only one of a wide variety of responses at any given brain site."

   This method is built around measuring cross-correlations of single responses rather than in the averages (ERPs), using the natural hierarchical structure enabled by repeated stimulations at the same brain site.

   c. More generally, other updated methods have been proposed for measuring CCEP responses (https://doi.org/10.1016/j.jneumeth.2019.108559, https://doi.org/10.1016/j.neuroimage.2017.07.061). The paper would benefit from engaging with basic, traditional methods and some of these updated methods and an explanation of why this newer method is more useful.
   Respectfully, we did not find traditional approaches to CCEP research to be relevant for our research questions, which is why this framework. The convergent approach and the BPC framework were designed to naively extract groups of electrodes that cluster together in terms of how they affect a recording site, and to discover this shape. Fitting

a set of pre-defined curves as suggestion in the cited Prime et al., 2020 is not. A graph theoretic approach as described in Krieg et al., 2017 might assist with understanding of hubs in a larger network, but these would be generally applied to an all-to-all approach, and are not oriented to capturing the full timecourses of responses in a way that satisfies the desired properties of analysis that we articulate.

d. One of the key differences in this method is that the authors do not average response to stimulation at a single site but examine response curves for all trials, which introduces some issues with shifts in baseline (as they acknowledge). They should make this clear when comparing their technique to others.

We would like to respectfully note that we do show averaged responses to stimulation at a single site, illustrated in the figure noted (Figure 2). Caption 2C now reads more clearly: "Biphasic stimulation pulses were delivered between adjacent electrodes throughout the array (gray shows all stimulation pulse trials for stimulation at each site, red shows average)." Caption 2E reads: "Averaged subgroup responses $G_n(t)$ (from the PHG measurement site) are shown between the two electrode sites that produced them"

We have modified the text to state this more clearly: "An example of potential non-independence may be seen in the non-zero offset of individual trials from the yellow site pair in Figure 2C, presumably due to direct charging of the cortical lamina from the prior stimulation pulse due to proximity of the stimulation-pair sites to the recording site."

e. As a point of clarification, is every stimulation trial mapped to every other stimulation trial in the initial step of the pipeline? Or just the first trial? This was not entirely clear to me from the figure.
The caption has been modified for clarity "Within stimulation-pair subgroup self-projections (all trials are projected into one another)."

2. The authors propose some uses of CCEP mapping but seem to ignore the principal application, which is understanding seizure networks (https://doi.org/10.1016/j.eplepsyres.2015.04.009).
    a. Readers considering application of the technique would be interested to know if it may have utility in this area.
    Our paper sets out to develop a fundamental method to understand convergent inputs to a specific brain region and it can be applied to fundamental neuroscience as well as applied clinical questions. We make the data and code available with the publication and did our best to fully describe the underlying mathematical approach such that this method allows other researchers to assess whether BPCs change in non-epileptic versus epileptic tissue. We have added new content to the "potential future applications" section of the discussion to address this: "Future work will explore this and will also examine whether specific BPC shapes are associated with similar biological motifs convergent on different brain regions (e.g. thalamocortical relays, U-fiber projections, or a common interneuron-projection neuron structure). While this work does not explicitly address biomarkers of disease state, one might hypothesize that seizure networks and

onset zones will have altered dynamics reflecting epileptogenicity, with corresponding abnormality in BPC shape.”

b. Related to this point, one of the issues with CCEP has been how to control for the effect of expected response magnitude due to anatomical distance (including volume conduction https://doi.org/10.1016/j.jneumeth.2020.108639). How does this new method handle this issue?

In the formalism for parameterization to quantify BPCs the response magnitude is independent of the shape. Because we take the cross projections between different trials, it is the reliability of the BPC shape across trials that drives the significance matrix that used for non-negative matrix factorization. It is important to consider that the data shown in our paper include the interval from 50-2000ms after stimulation. The largest effect of distance as noted in the cited work from Prime et al., focuses on the first 100 ms after stimulation. We have added the following text to our manuscript, citing the Prime et al manuscript, to reflect this: “For this illustration, \t_{1} \) was set to 0.050 sec and \(t_{2} \) to 2.000 sec, so as to begin after the majority of the volume conducted \& artifactural changes had passed \cite{prime2020quantifying}, and to extend until most responses have returned to baseline.”

c. Based on the data in Fig 4, it seems that significant response motifs are either nearby (green) or further away (yellow), but most sites near the electrode of interest do not show a significant BPC response.

d. Are strong responses not observed for nearby locations because the resulting responses are not consistent and end up being removed as noise? Or were they just not stimulated? Additional plots showing how the new method handles this issue would be a nice addition to the manuscript.

We would like to respectfully note that the data show the opposite of what the reviewer suggests. Namely, the yellow stimulation site is next to the electrode of interest (circled in red in figure 4, which is shown below for convenience), as is the orange, and these were the closest stimulated sites. Green is much further away. The figure is shown below.

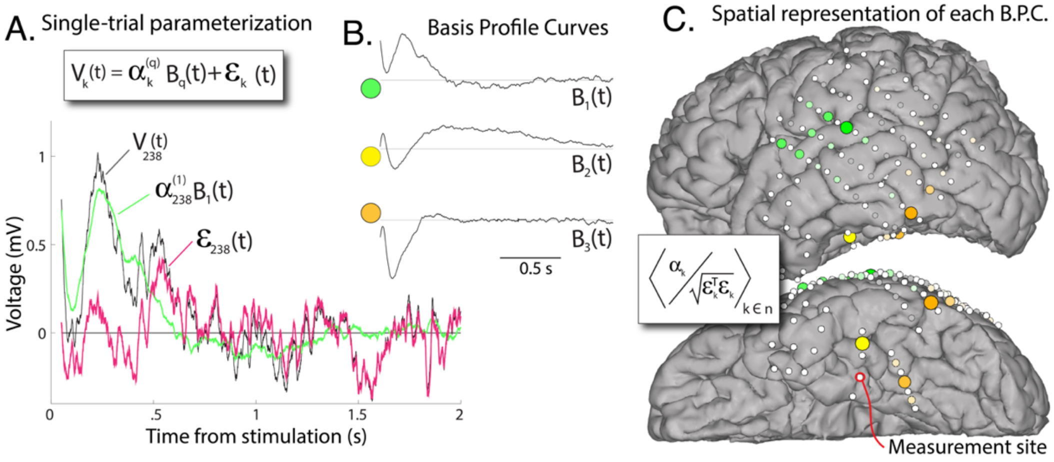

3. Related to this, additional explanation would be helpful in Fig 4. Most important is to show clearly where the measured response is located on the brain, the site to which the colored circles are presumably connected (what is the "convergent" electrode site?). This is more clear in the supplemental figure for temporal pole.
   We would respectfully like to note that, as is the case for the figure for the temporal pole in the supplement, the measurement site is circled in red and connected with a red line to text labeled "Measurement site" (as seen in the reproduced figure shown for convenience in the response above).

4. The authors include a paragraph describing the additional studies needed to verify the utility of this new method. I understand that this manuscript focuses on the methodological details. But for a high profile journal, I think a demonstration that this method identifies a connection of known anatomical effective connectivity (such as the arcuate fasiculus, visual-fusiform connections, as published for early validation of basic CCEP methods) would make the argument more convincing. Comparing how the BPC method performs compared to existing methods for such a known connection would be helpful.
   We would respectfully note that neither of these tracts are connected to the parahippocampal gyrus where our example data were recorded from. An analysis comparing CCEPs to known anatomic connections would be significantly different from the scope and purpose of this work. While it is an interesting scientific question, we would prefer to defer this to future studies. Our goal with this manuscript is to articulate this technique as clearly as possible and illustrate it with real data from one of our patients. We strongly believe that extending the scope beyond this would detract from the utility and impact of our work.

5. How do the authors handle multiple stimulation amplitudes? They report a single 6 mA threshold, but because of differences in impedance across stimulating locations (due to heterogeneity in electrode contact with the cortex), application of CCEP usually includes several stimulation thresholds (eg 2 to 8 mA).
   a. Why did the authors use only a single amplitude in their input data?
   b. They mention this as a future application, but I would assume several stimulation amplitudes are available for the example they show based on how CCEP data are collected. Do different stim amplitudes at the same region identify significant motifs (perhaps with different shapes after projection onto the observed response curves)?

      Due to limited time allotted for our experimental protocol, we have favored stimulation of many sites rather than many amplitudes in our recordings. We do not record multiple stimulation amplitudes. In the general sense, it otherwise lends analyses to the (N^2)^(number of stimulation levels) number of comparisons. We would ask that the reviewer respect that our present scope is our desired scope for the work we've done. The purpose of this work is to simplify the paradigm to something tractable, from which future studies at multiple levels might extend.
      - While we did discuss this in our manuscript previously, we have added further text to address this issue this further. From the last subsection of the discussion: "One example of this would be where evoked responses to electrical stimulation may vary based upon changes in region-specific ongoing fluctuations in neuronal excitability. If threshold levels must be met before specific components of a composite response are elicited (for example, a delayed second voltage deflection), then this method (which forces a unitized shape) would be sub-optimal and another strategy should be employed."

- The multi-amplitude approach could be studied in the hypothesis-preselected paradigm. In order to address this, we have added to and modified the text in the "potential future applications" section of the discussion: "For example, one could examine the effect of focusing on a pair of brain locations (hypothesis preselected paradigm) and simply changing the amplitude of stimulation. It has been demonstrated that different stimulation magnitudes can elicit very different morphologies (kundu 2020), and one could study this by labeling each stimulation amplitude as a different subgroup, and then examining the distribution of BPCs that emerge."

c. Finally, I don't know if this method would be useful in stereo EEG (depth electrode) data. The variation in the positive/negative deflection attributed to different interactions of depth electrodes with gray matter led to the adoption of techniques such as root mean square or gamma power to measure CCEP responses (https://doi.org/10.1016/j.cortex.2019.05.019). This same issue impacts the use of evoked potentials using stereo EEG recording electrodes. Based on the description provided in page 13, my sense is that the authors would say that this method is useful only for surface electrodes, where the relationship to the cortical surface is consistent, but perhaps this is incorrect. Given the explanation of how their method maps onto the neurophysiological properties of the brain, if the authors believe it would be useful in stereo EEG, showing some data to this effect would be important.
The convergent paradigm and the BPC approach are actually optimal for SEEG research because of the heterogeneous nature of the recordings. We state this in a new sentence of the discussion: "In particular, probing of the heterogeneous brain depths with stereoelectroencephalography appears to be a particularly useful application of the convergent paradigm, from which BPCs might be extracted."

6. Typos I noticed:
   a. Line 226, Fixed, thank you!
   b. Line 263, Fixed, thank you!

Reviewer #2: In this manuscript, Miller et al. outline a conceptual and computational approach to "convergent" brain stimulation mapping. They argue that, by stimulating many different sites and measuring the potentials evoked at a single target site (convergent approach), we generate a more interpretable dataset than by stimulating one site and measuring the resulting potentials at many other recipient sites (divergent approach). They further provide a computational approach for convergent mapping, based on a two stage algorithm: non-negative matrix factorization followed by linear kernel PCA. Their methods are illustrated with an example dataset and perform well in this setting.

Overall, this is an important, insightful and useful contribution to the theoretical framework around electrical brain stimulation. The main areas for improvement in the manuscript relate to the textual justification of the two-stage electrode-assignment and waveform-assignment approach, and clarification of some of the writing and presentation around the algorithm.

MAIN POINTS

1) The computational scheme outlined here seeks to satisfy two goals: (G1) identify groups of locations whose stimulation elicits similar waveforms at the target site, and (G2) to extract canonical waveforms (basis profile curves, BPCs) and associate them with individual stimulation events. There are conceptual and practical questions about this consecutive two stage approach.

Conceptually, it seems important to state explicitly what is the relative priority of each of these goals. In other words, suppose it were possible to achieve a better or more ambiguous solution to (G1) while rendering (G2) less interpretable or less accurate — which of these goals takes priority? You could imagine this as a kind of objective function in which we are optimizing for both goals — what is the weighting on each term in the objective function?
The reviewer raises an important issue. Using the G1/G2 convention of the reviewer:

- **(G1)** Our primary initial objective was to characterize whether there is structure in the response to stimulation at a particular brain site in response to stimulation at other sites. We made a simple observation that there are visually apparent clustering of responses from different stimulation sites, and this primary objective (G1 by the reviewer) represents an attempt to identify those clusters. That adjacent brain stimulation sites cluster together was quite interesting. This grouping is accomplished by generation of a 'significance matrix' (Figure 3D) that assesses similarity between individual events (trials), taking into account the sites of stimulation. The significance matrix is decomposed using NNMF, and using inner-dimension reduction to identify clusters of stimulation sites with similar responses.
- **(G2)** Equipped with this clustering, the extraction of canonical curve shapes (BPCs) is an attempt to find the structure in the response that is preserved across the cluster. This is a type of secondary objective. To get this structure, all trials clustered together were lumped together, with the label of where the stimulation came from removed. A PCA approach was used rather than a simple average of these trials so large deflections or noise on individual trials (that did not contribute to a shared variance) would be less influential on the obtained curve shape. Linear kernel PCA rather than standard PCA was necessary because of the limited trial number compared with the number of timepoints in each response.
- The individual trials may then be parameterized in terms of the BPC to which they are associated (e.g. the BPC associated with the cluster to which their stimulation electrode pair belongs). In some cases, the responses produced by a stimulation site may meet criteria to be

included in a cluster (e.g. by the thresholding following NNMF), but the parameterization reveals that they don't produce a strong response (one example would be the red-circled site below).

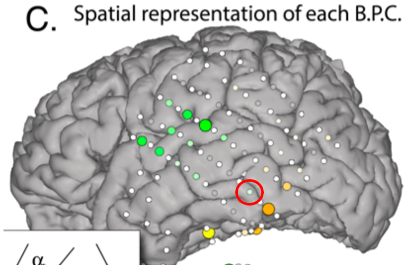

Practically, I could not help wondering whether a recursive procedure might not work better to jointly achieve the two goals (G1) and (G2). In many clustering settings [see for example, k-means clustering, but the principle applies more generally to all kinds of expectation-maximization algorithms] a broad approach is to (Step 1) make a preliminary clustering of trials and then (Step 2) extract canonical waveforms form each cluster, and then return to step (Step 1) using the canonical waveforms extracted in (Step 2) as a reference or basis of some kind. Conversely, in the current paper the two stages (clustering trials / electrodes, and then extracting BPCs) are performed strictly consecutively. Could the BPCs not be used as a means of performing better clustering? Do the authors think that the consecutive approach taken here is better than a recursive methods, or simply more straightforward?
In brief, we use a three-step approach in order to make the methodological insight/novelty more clear. The key elements of our approach are to generate a significance matrix that characterizes group-group similarity, to reduce this significance matrix (& thereby perform clustering), and then to recapture the structure that explained this similarity. We attempted to do this in the simplest way possible. Although there are more sophisticated approaches that could have been used for each step, we felt that would make the key components to capture how stimulation-pair subgroup cluster together more opaque. For example, linear kernel PCA is basically the simplest thing that we could do other than the simple average trace (which adds in a lot of noise)

The BPC approach we introduce could be incorporated into a recursive scheme, but our goal with this work is to introduce a framework to think about data of this type and make clustering tractable. We felt (and still feel) that this level of subtlety would overshadow our main purposes in this manuscript. This does not mean that a recursive approach will not ultimately be productive and may prove useful in the future (as we note in new text).

Application of a k-means approach to the set of all trials does recapture some of the structure (albeit noisier, and not as clearly representative of the stimulation-pair induced CCEPs as BPCs), as seen here:

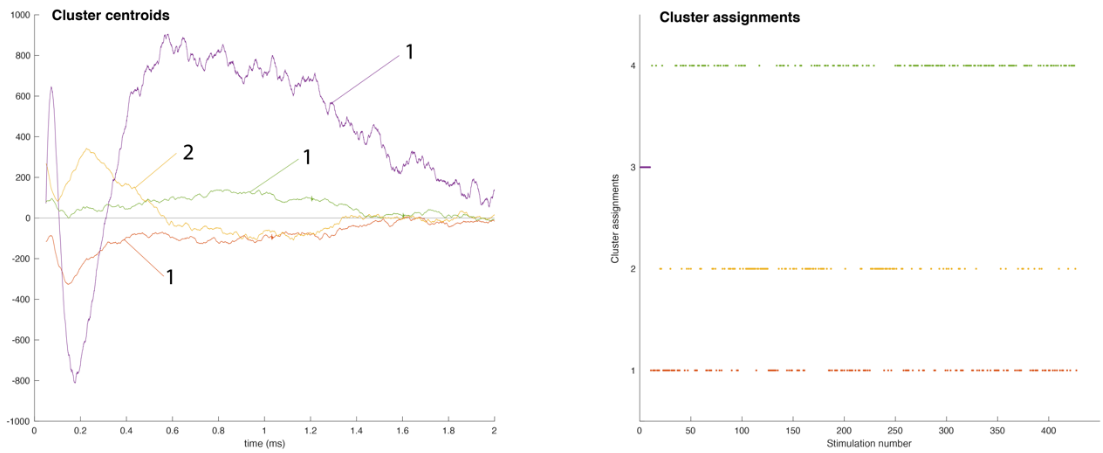

This introduces noise to the process because it does not exclude stimulation-pair subgroups that do not induce a response from the process of identifying canonical response shape. However, one could attempt to assign a cluster to each stimulation-pair subgroup by its predominant cluster assignment, and then rediscover a shape from the set of included stimulation-pair subgroups. Such an approach might be elaborated on and developed, but this undertaking would be a marked divergence from the present study (although we do mention it in new text).

We have summarized this discussion with the reviewer in a new paragraph added to the discussion subsection "Potential future applications" (first paragraph) that discusses the distinction in utility between the spatial distribution of clusters that induce a particular curve shape and the temporal form of that curve shape (this also incorporates the insight from major point 7 below): "The key elements of our approach are to generate a significance matrix that characterizes group-group similarity, to reduce this significance matrix (\& thereby perform clustering), and then to recapture the structure that explained this similarity. This is novel because it clusters group-labelled measurements (e.g. subgroups of repeated stimulations through same electrode pairs), discovering a hierarchical structure in the data. We have explicitly streamlined the framework in order to make the principles clear, and this process has been robust in all of the data we have analyzed. However, future opportunities may also be explored in other settings, adding complexity at each step. For example, we implemented linear kernel PCA to identify the BPC underlying a clustered set of trials – this is the simplest strategy other than the simplest average trace, which can add a lot of noise from weakly contributing subgroups. One could instead implement a dictionary learning approach and use the atom that explains the most variance of the included trials \cite{mairal2009online}. The sequential approach that we use for methodological clarity might instead be expanded into a recursive approach. In this setting, discovered BPCs would be used to re-parameterize the initial trials, these parameterizations would then be clustered, and a new set of BPCs would be found, iterating the process until convergence criteria are met. Alternate approaches to achieve hierarchical clustering with known subgroup labels might begin with a common technique applied to all trials (e.g. k-means, Gaussean mixture model, etc), and then associate these clusters with stimulation-pair subgroups. We have deferred presentation of these types of analyses because each would involve a study of the same scope as the present one and distract from the key core elements of the BPC framework."

2) A key assumption of the decomposition (lines 63-65) is that each stimulation trial should be

characterized by a single BPC rather than a superposition of BPCs. I would like to hear more about whether this is a computationally expedient assumption, or whether it is endorsing an assumption about the nature of the mapping process and brain dynamics.
Rather than computational expedience, the reason for associating a single BPC with each stimulation pair (and thus each stimulation) is *cognitive* expedience. Our biggest struggle with this type of CCEP/SPES analysis has been simplifying it to something we can "wrap our heads around" (e.g. tractable). As a first order approach, we need to have this simple association in order to explore networks (otherwise, we have found this problem intractable). It says more about how we do research than about the brain, but it does have constraints. In order to address this, we have added and modified the text in the "future directions" section of the discussion, in the paragraph that begins 'This "winner-take-all" approach…: "One example of this would be where evoked responses to electrical stimulation may vary based upon changes in region-specific ongoing fluctuations in neuronal excitability. If threshold levels must be met before specific components of a composite response are elicited (for example, a delayed second voltage deflection), then this method (which forces a unitized shape) would be sub-optimal, and another strategy should be employed."

In particular, it seems that if there are ongoing fluctuations in the excitability of the neurons in the target site, then this alone could give rise to variation in the trial-to-trial mixture of responses elicited in a single site. For example, consider the case where we are always stimulating exactly the same adjacent electrode-pair and recording from the same target site. Now, suppose that one BPC is associated with "high pre-stim excitability of pyramidal neurons at the target site" and another is associated with "low pre-stim excitability fo pyramidal neurons at the target sites"… would one not expect that a superposition of these BPCs would be observed on stimulation trials on which the pre-stimulation excitability in the target site is "intermediate excitability"?
The reviewer raises an important point: evoked responses to electrical stimulation may vary based upon changes in region-specific ongoing fluctuations in neuronal excitability. If threshold levels must be met before specific components of a composite response are elicited, then this method (which forces a unitized shape) would be suboptimal. In order to address this, we have added content to the last subsection of the discussion: "One example of this would be where evoked responses to electrical stimulation may vary based upon changes in region-specific ongoing fluctuations in neuronal excitability. If threshold levels must be met before specific components of a composite response are elicited (for example, a delayed second voltage deflection), then this method (which forces a unitized shape) would be sub-optimal and another strategy should be employed."

3) At a broader level, I would have liked to have seen a few sentences in the Discussion addressing the broad question: "How would we know if the results of this method are better than another method? What should we treat as ground truth, or the practical outcome which can be used to decide that one decomposition approach is ideal"? I could imagine pure neuroscience arguments here (e.g. consistency in extracted clusterings when using convergent mapping for target site A and target site B) and more applied outcomes (e.g. better performance in identifying pathological brain tissue). Ultimately, though, I think that a few sentences on this topic could help to remind the reader what all of these methods are for, and to provide a framework for comparing these methods against any other approaches.
We have added new content to the "potential future applications" section of the discussion to address this: "Future work will explore this and will also examine whether specific BPC shapes are associated with similar biological motifs convergent on different brain regions (e.g. thalamocortical relays, U-fiber projections, or a common interneuron-projection neuron structure). While this work does not explicitly address biomarkers of disease state, one might hypothesize that seizure networks and onset zones will have altered dynamics reflecting epileptogenicity, with corresponding abnormality in BPC shape."

4) For selecting the BPCs (paragraphs around lines 162 and 180), is the selection based on explained variance ideal, or could it be improved using a cross-validation based selection procedure? [Hold out some of the data; fit the model; measure the goodness of reconstruction in the held-out data]. The authors need not implement this, but out-of-sample explained variance may be worth mentioning as an alternative to in-sample explained variance.

One could incorporate an element of cross-validation into the approach, but that requires a layered parameterization at the beginning of the process. It would markedly expand the scope of the manuscript, and we are concerned that this would be at the expense of the clarity of the key elements (as discussed above). Nonetheless, we do believe that the reader should be made aware of this distinction. In order to address this explicitly, we have added and modified the text (in a new paragraph added to the "potential future applications" section of the discussion): "The sequential approach that we use for methodological clarity might instead be expanded into a recursive approach. In this setting, discovered BPCs would be used to re-parameterize the initial trials, these parameterizations would then be clustered, and a new set of BPCs would be found, iterating the process until convergence criteria are met"

We have addressed the overall concept of cross-validation by illustrating the robustness of the technique with a 50-50 split of trials, illustrated in a new supplemental figure, and referenced in the main text of the Results section: "The technique produces an intuitive representation of the responses measured at a single site in the convergent paradigm, and it is robust. Splitting the data in half yields two similar sets of BPCs, even though there are only 4-5 trials per stimulation-pair subgroup (Supplemental figure 4)."

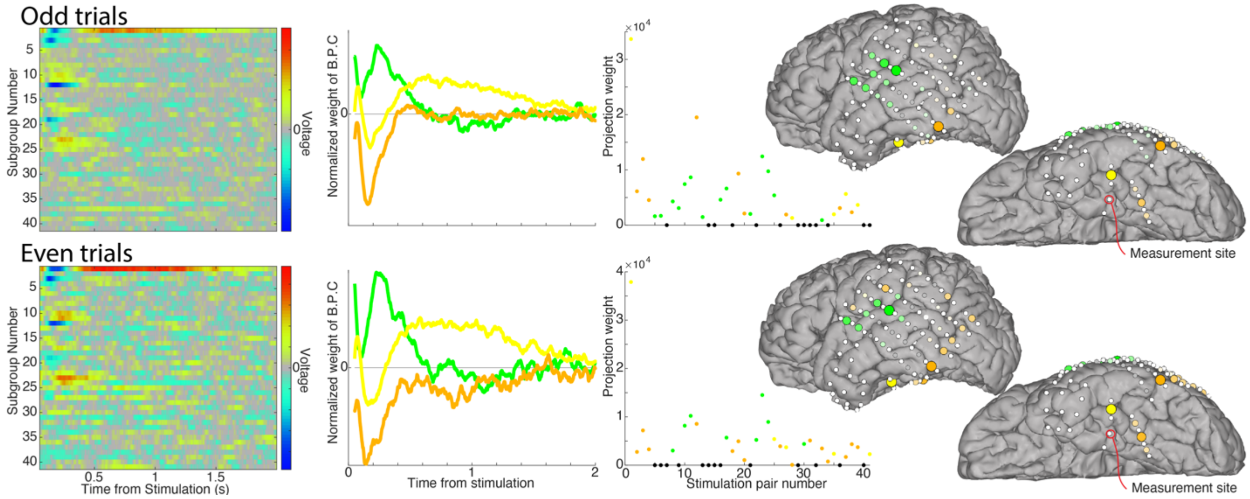

"Supplemental Fig 4. An illustration of the BPC method applied to sub-segmentation of trials. On the top row, BPCs are identified from only odd trials. On the bottom row, BPCs are identified from only even trials. After the split, each stimulation-pair subgroup is represented by 4-5 individual stimulation trials. Despite this small amount of individual stimulation trials for each stimulation-pair subgroup, the process is remarkably stable. For each row, the panels from left-to-right are matrices of the CCEPs, extracted BPC shapes, projection weights (group-averaged signal-to-noise ratio), and projection weights plotted on the brain surface."

5) Figure 4: Could the authors comment on why the ventral visual system is so responsive to stimulation in the ventral motor cortex? Obviously resolving this is not important for this manuscript, but it seems worth commenting on the spatial distribution shown in Figure 4C, in light of the very ventral site (PHG) that is being targeted.
It is an artifact of the way the data are illustrated that make it appear that way (i.e. stimulation pairs that flank the sylvian fissure appear to be closer to the superior side of it – near the ventral paracentral lobule / post-central gyrus). The response is from the superior temporal gyrus. In order to clarify this point more clearly, we have added the following text to the second paragraph last subsection of the results: "Note that the most superior sites included in the STG-BPC may not necessarily result from projections above the Sylvian fissure (e.g. motor areas) to the PHG, but likely result from one of the two electrodes in the included stimulation pair lying on or below the Sylvian fissure."

6) Line 292: "We found instead that the measured cortico-cortical evoked potentials could be described by three basis profile curves, which are unique in shape (Figure 4B), only one of which (B3) is consistent with the reported N1/N2 form. " This seems like an important point — at the same time, when making an observation like this it seems important to confirm for the reader that the N1/N2 forms are "ostensibly" present in the raw data. Because another reason why they may not be recovered by this procedure could be that they are simply not present in this example dataset… so you would want to show that the N1/N2 components //are// (under some conventional appraisal) present in the raw data (perhaps this is just done by eye?) and yet these N1/N2 peaks are not recovered as BPCs.
Unfortunately, the N1/N2 components are often not present in the raw data, as can be seen in the matrix of the raw responses in figure 2D and 2E. They are also often not seen in measurements from another site (Supplemental figure 3) in the same patient. In recordings from another institution from primary motor cortex, we also frequently did not see it[1]. We also don't see it in the raw data from other brain areas in recordings from this patient, as seen in the figures below.

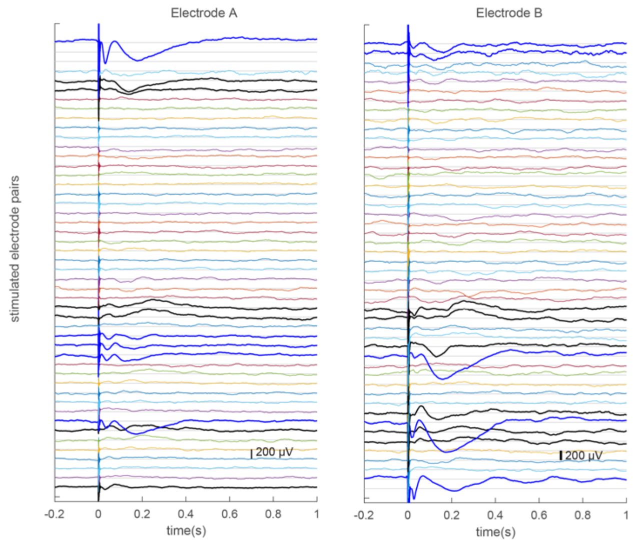

This figure shows all CCEPs from two sites, without excluding the peri-stim time. Note the heterogeneity in response, which does not universally show an N1/N2 form.

[1] Miller KJ, Huiskamp G, van Blooijs D, Hermes D, Gebbink TA, Ferrier CH, et al.An observation of anatomical clustering in inputs to primary motor cortex incortico-cortical brain surface evoked potentials. In: 2019 7th International WinterConference on Brain-Computer Interface (BCI). IEEE; 2019. p. 1–2.

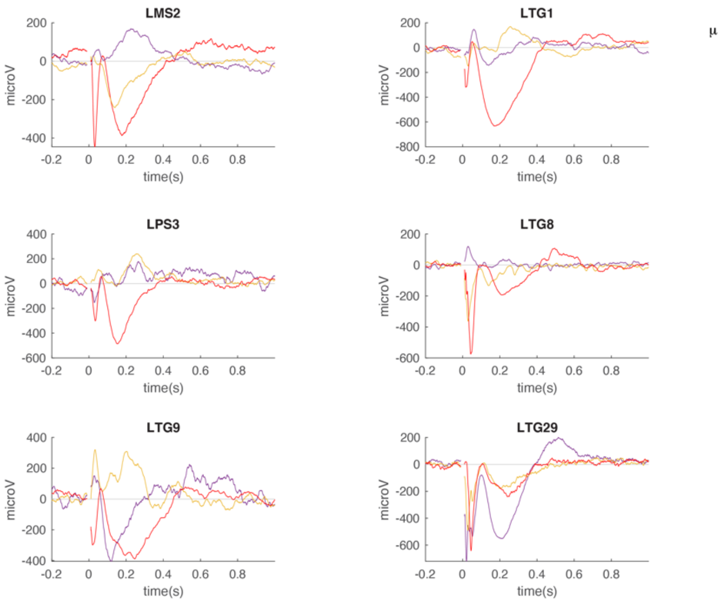

This figure shows 3 significant, distinct, CCEP shapes measured from 6 sites in our example patient. While the N1/N2 is found (red traces), other unrelated shapes are seen as well.

In order to more clearly emphasize this, we have modified the text of the introduction with a sentence that reads: "Notably, we have found that the N1/N2 response shape is not a universal phenomenon: when observed, this shape is only one of a wide variety of responses at any given brain site."

7) Although I find the arguments in favor of the "convergent" mapping procedure compelling (as opposed to divergent), I think that what the authors present here — mapping of /two/ sites using convergent mapping and then comparing the maps — is even more compelling, because it enables you to see whether the same site-clusters (e.g. the motor cortex cluster) are identified as clusters for two different target sites. If the authors agree, then they may want to consider moving Supp Figure 3D/E into the main text?

Our goal with this manuscript is to articulate this technique as clearly as possible and illustrate it with real data from one of our patients. While we agree that showing overlapping spatial motifs from BPCs identified from different convergent sites is a compelling direction to move into, we are concerned that elevation of this to the main text would expands the scope of the manuscript in a direction we are not comfortable with, and distracts from our initial primary goals. Work in progress will clearly articulate this phenomenon, across a broad range of patients for specific functional domains. This will be deferred to future work, but we do think it could be more clearly hinted at, and we have added new text to reflect this: "One might speculate about different types of connectivity within the temporal lobe that may be inferred from examination of figures 4 and S5: The preliminary finding of similar spatial distribution in a BPC from the STG, but reflected with a different temporal shape in the TP than the PHG suggests different kinds of projections emanating from a well-defined STG region. For example, direct connections from intracortical axonal projects within gray matter (via lateral projections) might be differentiated from those relayed subcortically through white matter tracts. Note that indirect projections relayed through a third cortical site or a set of subcortical nuclei might each be revealed in characteristic BPC shapes, and this will be explored in future studies."

……

MINOR POINTS

0) Throughout the manuscript "figure 2" and "figure 3" references could be "Figure 2" and "Figure 3".
We have changed this throughout. Thank you.

1) Might sparse dictionary learning methods be used in place of the linear kernel PCA approach? (e.g. Mairal et al., 2009, Online Dictionary Learning for Sparse Coding. ICML).
This is possible to do in principle. However, as we began to explore this, we found that it was significantly expanding the scope of the manuscript and detracting from the core elements that we find most critical to try to communicate. However, we do feel that this is important to make the reader aware of. As such, we have added the following text to the new paragraph in the discussion: "… we implemented linear kernel PCA to identify the BPC underlying a clustered set of trials – this is the simplest strategy other than the simplest average trace, which can add a lot of noise from weakly contributing subgroups. One could instead implement a dictionary learning approach and use the atom that explains the most variance of the included trials \cite{mairal2009online}."

2) In Figure 2, the site of each stimulation site is mapped on the cortical surface. Is this "site" of stimulation the spatial average of the positions of the two stimulated electrodes?
In Figure 2E, the axes are placed in between the positions of the two stimulated electrodes.
We have modified the text of the caption to make this clearer: "Averaged subgroup responses $G_n(t)$ (from the PHG measurement site) are shown between the two electrode sites that produced them."

In Figure 4, the mapped circle is, in fact, the spatial average of the positions of the two stimulated electrodes (as the reviewer notes). We have modified the text of the caption to reflect this, adding: "White circles show actual electrode locations and BPC projection magnitudes are placed at the the spatial average of the positions of the two stimulated electrodes."

Line 17: this sentence seems awkward toward the end:
"For an array of N total electrodes, there are a potential set of order N2 CCEP interactions that may be explored (for bipolar stimulation, the actual number will depend on which sets of adjacent stimulation pairs are chosen to deliver electrical pulses through)."
should "stimulation pairs" be "electrode pairs"?
It has been changed for clarity to now read "For an array of N total electrodes, there are a potential set of order N^2 CCEP interactions that may be explored (for bipolar stimulation, the exact number will depend on how neighboring electrode pairs are chosen for stimulation)."

Line 27: "and then study" could be "and then to study"
Thank you for identifying this. The text has been changed to read "and then to study".

Line 101: "T total time points" could be "T time points"
We respectfully wish to keep it as "T total time points" to remain parallel with "K total stimulation events"

Line 103: May want to make this more concrete for the reader by saying something like, "In the example provided here, each subgroup corresponds to a pair of adjacent electrodes"
Thank you for the recommendation. "In the example provided here, each subgroup corresponds to a pair of adjacent electrodes that are stimulated between."

Line 120: the notation in the equation for S_{nvm} is confusing , with k an element of n, and l an element of m… if n and m are sets, would it not be clearer to demarcate them with capital letters or some other token?

The notation for "n" comes from an established subgroup label in the section above, with the capital letter "N" already in use for the total number of subgroups. While re-reading our text, we do agree with the reviewer about the need to clarify. In order to clarify, we have modified and added to the text, which now reads:

"The full matrix **P** is subsequently sorted into an array of sets $S_{n,m}$ that characterize each cross-subgroup interaction (e.g. pairwise interaction between subgroups *n* and *m*), such that:"

Line 124: this matrix is first subjected to a non-negativity constraints — please clarify if "first" here means before or after t-value conversion… presumably it would have to happen after the t-value conversion? In that case it would not be the first manipulation? Maybe "next" rather than "first"?

We have changed it to read more clearly: "This matrix is then subjected to a non-negativity constraint, setting negative t-values to zero (a property that is needed for subsequent factorization):".

Line 132: "there is structure in the variation itself" — maybe this would be clearer as "there is reliable temporal structure in the within-group variation" ..?

"This occurs when there is reliable structure in the response, but, first, within-group variation is larger than the cross-group variation, and, second, there is reliable across-group temporal information across groups (for example, as result of sequence)."

Line 156: "pushing the normalization factor onto the columns of W" — please clarify

This has now been clarified to read: "Second, the rows of **H** are unit normalized, maintaining the overall scale by multiplying the columns of **W** by the the normalization factor:"

Line 159: the previous steps were written in passive tense and this is now in an imperative tense ("calculate" vs. "is calculated")

Thank you for identifying this. It now reads: "Fourth, the error is calculated as … and is assessed for convergence, …".

Line 182: I think this could be "it is more expedient to start with…"

The typo has been corrected. Thank you for identifying it.

Line 193: please clarify where the tolerance factor was added, and the sensitivity of the procedure to the choice of this parameter

I have changed the text so that it reads more clearly (retaining Latex embedding): "At the conclusion of this process, we perform a winner-take-all operation on the columns of **H** such that one stimulation-pair subgroup can only belong to one component (all but the largest elements of each column are set to zero).

For each row *q* of **H**, a set of stimulation-pair subgroups is assigned to each cluster by thresholding: If $ H_{q n} > \frac{1}{2\sqrt{N}} $, then $n \in q$. This threshold is set because if all subgroups contributed equally to a cluster, then the element weight of each would be $1 / \sqrt{N}$ (the tolerance factor of 1/2 allows for variation)."

For the example dataset, the choice of tolerance factor has minimal effect on the outcome of the process, as illustrated here:

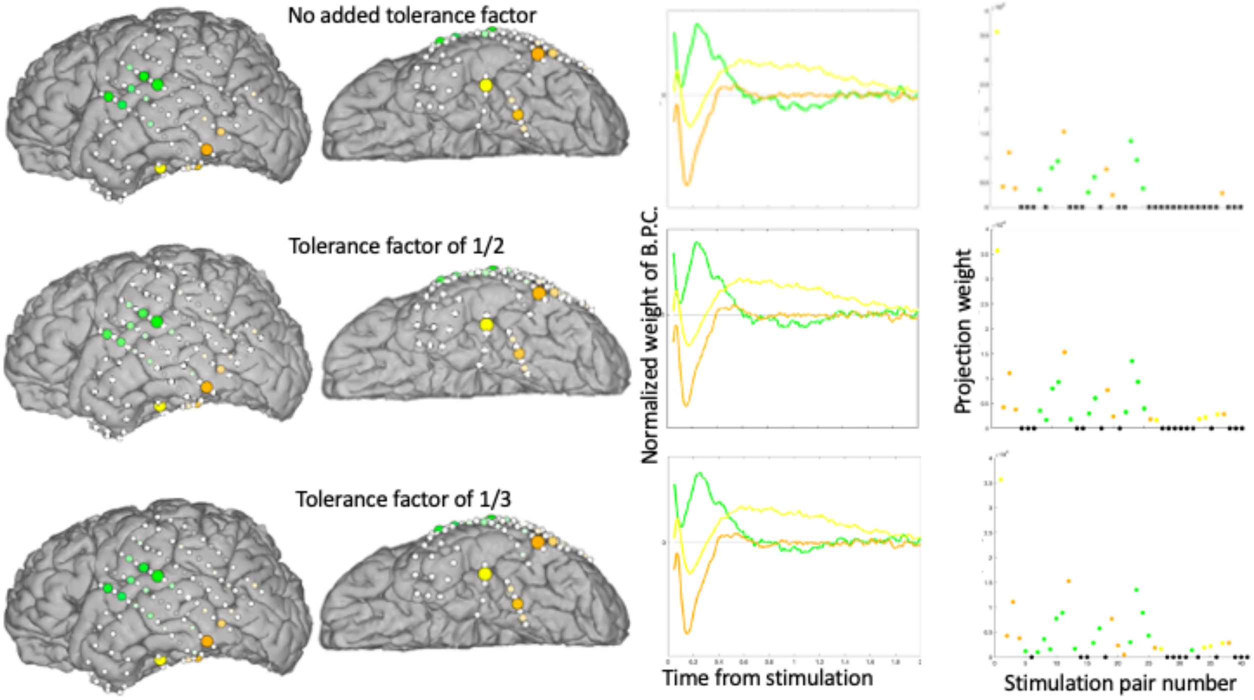

Line 226-7: "several insignificant stimulation-pair subgroups" — please make clear what "insignificant" means in this context — is this a statistical statement or estimate?

It is a qualitative statement. We have modified the text to indicate this explicitly and read more easily: "As illustrated in cluster 3 of our example, several low-relevance stimulation-pair subgroups (i.e. very low average projection weight) are clustered along with a strong and significant response subgroup. As we have constructed it, the kernel PCA approach captures the shape of this robust subgroup, without disruption from the uncorrelated noise that dominates the low-relevance subgroups."

Line 236: please check punctuation here … the structure of the sentence is "Since X, and Y=W=Z"

Thank you for identifying this. We have changed the grammar to make the calculation process clearer, as follows:

$$V_k(t) = \alpha_k^{(q)} B_q(t) + \varepsilon_k(t)$$

We expect that $E(\varepsilon) = 0$ and $E(\varepsilon_k^2) \sim E(\varepsilon_l^2)$, for all k and l. This allows us to estimate the projection of $B_q(t)$ into each individual trial as follows. First, we expand our single-trial formalism above by application of $\sum_t B_q(t)$ to both sides, i.e.:

$$\sum_t B_q(t) V_k(t) = \sum_t B_q(t) \alpha_k^{(q)} B_q(t) + \sum_t B_q(t) \varepsilon_k(t)$$

However, $\sum_t B_q(t)\varepsilon_k(t) = 0$ since $E(\varepsilon) = 0$, and $\sum_t B_q(t)\alpha_k^{(q)} B_q(t) = \alpha_k^{(q)} \sum_t B_q(t) B_q(t)$, which is just $\alpha_k^{(q)}$, since $\sum_t B_q(t) B_q(t) = 1$. This allows us to calculate $\alpha_k^{(q)}$ for each trial:

$$\alpha_k^{(q)} = \sum_t B_q(t) V_k(t)$$

Line 250 : disentangling of this convergence —> disentangling this convergence
Fixed, thank you!

Line 255: based upon where was stimulated —> based on the stimulation site
Fixed, thank you!

Line 268: a single site —> at a single site
Fixed, thank you!

Line 279: "by their relative weights" — please specify which parameter this refers to
Thank you for identifying this. We have modified the text to now read: "The iterative reduction clusters stimulation-pair subgroups by their relative element sizes within the rows of the NNMF weight matrix **H**"

Line 358: "Although there is no prior assumption about the forms the BPCs should have, …" — perhaps specify by mentioning e.g., continuity or differentiability or variance or something like that… certainly the decomposition scheme must impose /some/ assumptions and constraints on the form of the BPCs… it is just a fairly generic time-series decomposition assumption… I would imagine that this matrix decomposition technique could be re-written as a kind of Bayesian inference procedure, with some priors over the variables, correct?
There are no specific assumptions about the form of the BPCs. For illustration, consider the 2 following "model" response profiles, one of which is not continuous, and the other of which is not differentiable, and each has very different variance from the other:

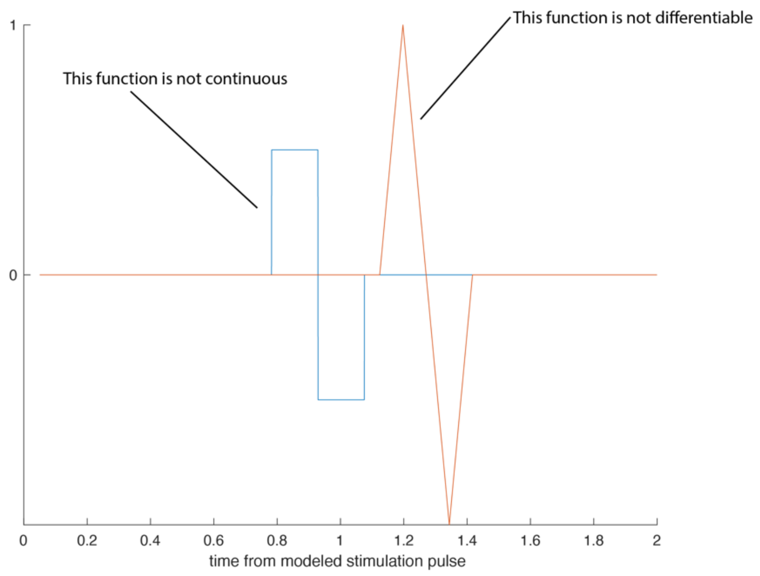

These are then used to create model subgroups (4 of 10 for each type) that are scaled randomly and individually, and then a small amount of continuous noise is added. Plotted as in Fig 2D.

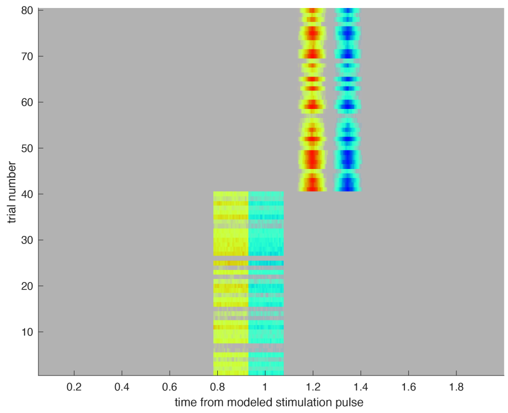

These “trials” are added to the native data from the example case:

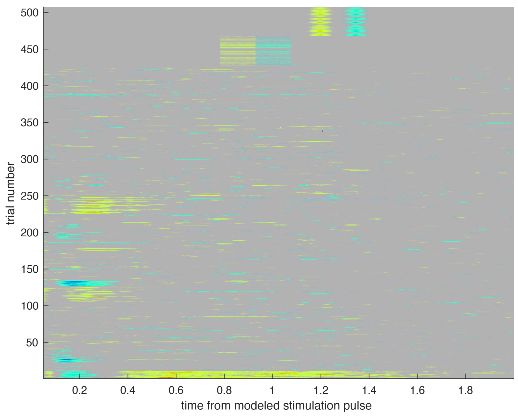

The BPC process is then run on these data, recovering the original BPCs, as well as the new “synthetic” ones.

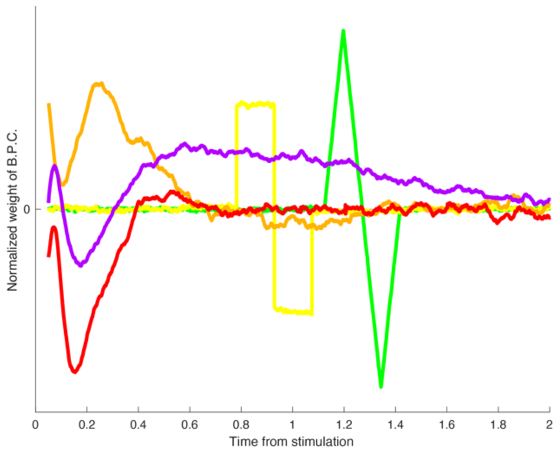

This MATLAB script necessary to reproduce this illustration is as follows:

```
%%%%%%%%%%%%%%%%%%%%%%%%%%%%%%%%%%%%%%%%%%%%%%%%%%%%%%%%%%%%%%%
%% generate base shapes of not differentiable and not continuous
    a=zeros(length(t_win),1); b=a;
    c=.5*(1+sawtooth([1:300]/(300/(2*pi)),.5));
    a(1501:1800)=.5; a(1801:2100)=-.5; b(2201:2500)=c;b(2501:2800)=-c;
```

```
    figure, plot(t_win,[a b]),xlabel('time from modeled stimulation pulse'),
    box off, set(gcf,'color','w'),set(gca,'ytick',[0 1])

%% create model subgroups (4 of 10 for each type that can be addended to matrix V)
    sf=1000; % scalefactor

    for k=1:40
        a_mat(:,k)=sf*((.25+rand)*a+.025*movsum(rand(length(t_win),1)-.5,15));
        b_mat(:,k)=sf*((.25+rand)*b+.025*movsum(rand(length(t_win),1)-.5,15));
    end

    V0=[a_mat b_mat];
    figure, kjm_imagesc(t_win,[1:80],V0),
    xlabel('time from modeled stimulation pulse'),
    ylabel('trial number'), box off, set(gcf,'color','w'),colormap(cm)

%% addend to matrix of trials (V), and create new "subgroups"
    V0=[V a_mat b_mat];

    figure, kjm_imagesc(t_win,[1:507],V0),
    xlabel('time from modeled stimulation pulse'), ylabel('trial number'),
    box off, set(gcf,'color','w'),colormap(cm)

    pair_types0=pair_types;

    pair_types0(42).pair=[101 102];
    pair_types0(42).indices=[428:437]';

    pair_types0(43).pair=[103 104];
    pair_types0(43).indices=[438:447]';

    pair_types0(44).pair=[105 106];
    pair_types0(44).indices=[448:457]';

    pair_types0(45).pair=[107 108];
    pair_types0(45).indices=[458:467]';

    pair_types0(46).pair=[201 202];
    pair_types0(46).indices=[468:477]';

    pair_types0(47).pair=[203 204];
    pair_types0(47).indices=[478:487]';

    pair_types0(48).pair=[205 206];
    pair_types0(48).indices=[488:497]';

    pair_types0(49).pair=[207 208];
    pair_types0(49).indices=[498:507]';

%% perform BPC identification
    [B_struct0,excluded_pairs0]=bpc_identify(V0,pair_types0);

    %% plot BPC traces - like in fig 4B, but colored
    traces_fig=figure;
    plot(t_win([1 end]),[0 0],'color',.5*[1 1 1]), hold on
    for q=1:length(B_struct0)
        figure(traces_fig)
        hold on, plot(t_win,B_struct0(q).curve,'color',maxcols(q,:),'LineWidth',3)
    end, clear q
    set(gca,'YTick',0),
    xlabel('Time from stimulation'), ylabel('Normalized weight of B.P.C.')
    set(gcf,'Name','Basis Profile Curves'), box off, set(gcf,'color','w')
%%%%%%%%%%%%%%%%%%%%%%%%%%%%%%%%%%%%%%%%%%%%%%%%%%%%%%%%%%%%%%%%
```

Line 446: “Each region is defined by a unique laminar architecture (defined as a unique Brodmann area, [32]),…” — Is this actually true? My understanding was that cytoarchitectonic features would sometimes vary in a graded manner, and that regional boundaries are drawn based on multiple features, including cortical thickness and myelination and other factors? This is a very small point… it just seems strange to

cite Korbinian Brodmann for this claim … maybe consider citing a more modern atlas, too, like some of the recent Van Essen group atlases based on multimodal imaging?

In our opinion, Brodmann was the first to really demonstrate unique laminar architecture, so we decided to cite that. We do agree that a more modern approach would also be appropriate to include, and have cited the Van Essen group as such. It now reads: "Each region is defined by a unique laminar architecture (defined histologically as a unique Brodmann area [32], or by multi-modal imaging as a unique parcel [33]), …".

Reviewer #3: I would like to commend the authors for their submitted manuscript, in which they clearly outline a novel way to cluster data resulting from medical singe pulse electrical stimulation (SPES) sessions in intracranially implanted patients. Indeed their approach appears to provide a data-driven way to quantitively tease apart different response profiles resulting from stimulation throughout the brain, which they call basis profile curves" (BPCs). This method provides clusters that cannot be obtained to more commonly employed methods (for example ICA, direct PCA based methods etc), which would probably cluster the 3 BPCs categorized in the example into the same cluster (possibly even producing a traditional N1/N2 profile), which would not reflect the single trial-level accurately. This method allows for data resulting from SPES sessions to be analysed in more depth and with higher accuracy, while still constraining the amount of variables. I am enthusiastic about this method and can see many potential applications in future analyses. The manuscript is very well-written and surely will benefit iEEG research.

I have only two concerns, one major and one minor (and list a few typos). I hope these help the authors to improve on the manuscript.

Specific comments

1. Major. The proposed approach will work very nicely when one considers only one electrode where signals are recorded. However, in many cases a researcher will have several electrodes in a given area. For instance, one may have 5 electrodes in the hippocampus and uses SPES data to test how the hippocampus is connected to other brain regions. This problem would even be more exacerbated when one has microwire recordings, and increasingly popular tool, where there are 8 channels per location. The approach presented by the authors would provide a handful of BPCs per channel, but how can we group these BPCs across electrodes in a meaningful way? Notably, when grouping across electrodes, phase-reversals (ie. polarity flips) are possible (even expected) so the NNMF wouldn't work in this instance. It would be useful if the authors could adapt their toolbox to implement such a second-level clustering solution. However, the authors may wish to answer that this question is beyond the scope, which is fair enough, but then at least they should offer some guidance as to how BPCs across electrodes can be grouped.
The reviewer makes an excellent point, and we do feel that many studies of BPCs should branch out from the single convergent measurement site, comparing responses from different contexts. A full treatment of this would be beyond our desired scope, but we have added text to point the reader along this direction in their own studies, in the "Potential future applications" section of the discussion:
"Natural branching out from the single seed site of the convergent paradigm will lead researchers to examine the generalization of BPC shapes discovered at adjacent measurement sites within a brain region, and will also lead them to explore whether they are conserved at homologous brain sites across different patients. Such "second-level" studies of BPC shape across contexts would employ more traditional clustering approaches than the present strategy. For example, measurements of the brain's depths with stereoelectroencephalography should allowance of sign flips when comparing BPCs from adjacent electrodes spanning superficial vs deep cortical layers."

2. Minor. This point relates to the brute-forcing of the non-negative matrix factorization (NNMF). You mention that alternative implementations to repeatedly re-running the NNMF were deemed impractical. I would like to see more detail about why that is the case. Is this due to intense computation requirements/times, that offset the advantage of reducing the randomness of W and H? I was also wondering how much variability this method leaves in resulting BPCs in data that are a bit more noisy/not as clear cut.
Using a minimization algorithm that varies the initial values for W and H (rather than selecting random

matrices), with final \zeta as an objective measure is not trivial. The mechanism for performing non-negative matrix factorization described in the sub-subsection "NNMF multiplicative update rules" uses a set of update rules that iteratively converges to minimize \zeta. Therefore, techniques to vary initial values of W and H while re-running NNMF would have to do so in a way that was, by construction, independent of the update rules intending to accomplish the same objective (minimization of \zeta). A gradient descent approach in generation of W and H, as a function of \zeta, is likely to recapitulate the structure of the NNMF. Some potential alternate approaches to do this would be a type of grid search across randomization seeds in generation of W and H, but where the search process was intentionally not smooth across the grid.

The reason we do not do this actually is the opposite of intense computational requirement, quantification times for the BPCs in our studies have been quite short (<3mins). A minimization algorithm for picking the seeds of NNMF introduces more complexity to an already complex process, so it is more practical to (potentially) use more computational time that is only of order a few minutes in the brute-force approach.

In order to clarify this, we have added and modified the text to read (in the "NNMF multiplicative update rules" section of the methods): "Separately, one might perform an algorithmic minimization with convergence rather than brute-force repetition, though we found this impractical for two reasons. First, calculation times for re-running the algorithm were quite short in our studies (<3 minutes), and second, approaches to re-parameterizing **W** and **H** for iterative convergence would require significant methodological treatment of their own."

Typos:
Line 144: … anatomy IS not …
Fixed, thank you!

Line 268: … observed AT a single site …
Fixed, thank you!

Line 435: there is a ')' missing in the reference to suppl fig 2.
Fixed, thank you!
